# Prostate carcinoma presenting with diffuse osteolytic metastases and supraclavicular lymphadenopathy mimicking multiple myeloma

**DOI:** 10.1002/ccr3.1336

**Published:** 2017-12-19

**Authors:** Bukunmi Michael Idowu

**Affiliations:** ^1^ Department of Radiology Obafemi Awolowo University Teaching Hospitals Complex Ile‐Ife Osun State Nigeria

**Keywords:** Bone, metastases, osteoblastic, osteolytic, prostate cancer

## Abstract

Prostate cancer typically presents with osteoblastic metastases; however, patients with prostate cancer may have osteolytic metastases, as seen in this case. Patients with osteolytic metastases require a thorough evaluation to rule out malignancies including multiple myeloma, but prostate cancer is a very important differential diagnosis.

## Introduction

Prostate cancer is more common in black men than men of other races 1. Osteoblastic metastases are frequently seen in prostate cancer, but diffuse osteolytic metastases are very rare 1, 2. This is a case of widespread bone metastases from prostate cancer in an elderly man.

## Case Presentation

A 66‐year‐old man presented with poor urinary stream, left supraclavicular swelling, and low back pain of less than 5 months’ duration. Digital rectal examination revealed an enlarged, tender, nodular prostate gland with deepened lateral sulcus, and obliterated median groove which was adherent to the overlying rectal mucosa. There was tenderness over the T11 – L1 vertebrae, but no obvious deformity or sensory deficit pattern was detected. An enlarged, nontender left supraclavicular lymph node measuring 3 cm × 3 cm was palpated. It was firm, smooth, and mobile.

The prostate‐specific antigen (PSA) was 16 ng/mL (Normal = 0–4 ng/mL); the erythrocyte sedimentation rate was also elevated (50 mm/h Westergren). Other laboratory tests were normal—there was no anemia, leukopenia, thrombocytopenia, hypercalcemia, or Bence‐Jones proteinuria. Renal function was normal as assessed with serum electrolyte/urea/creatinine test.

**Figure 1:**
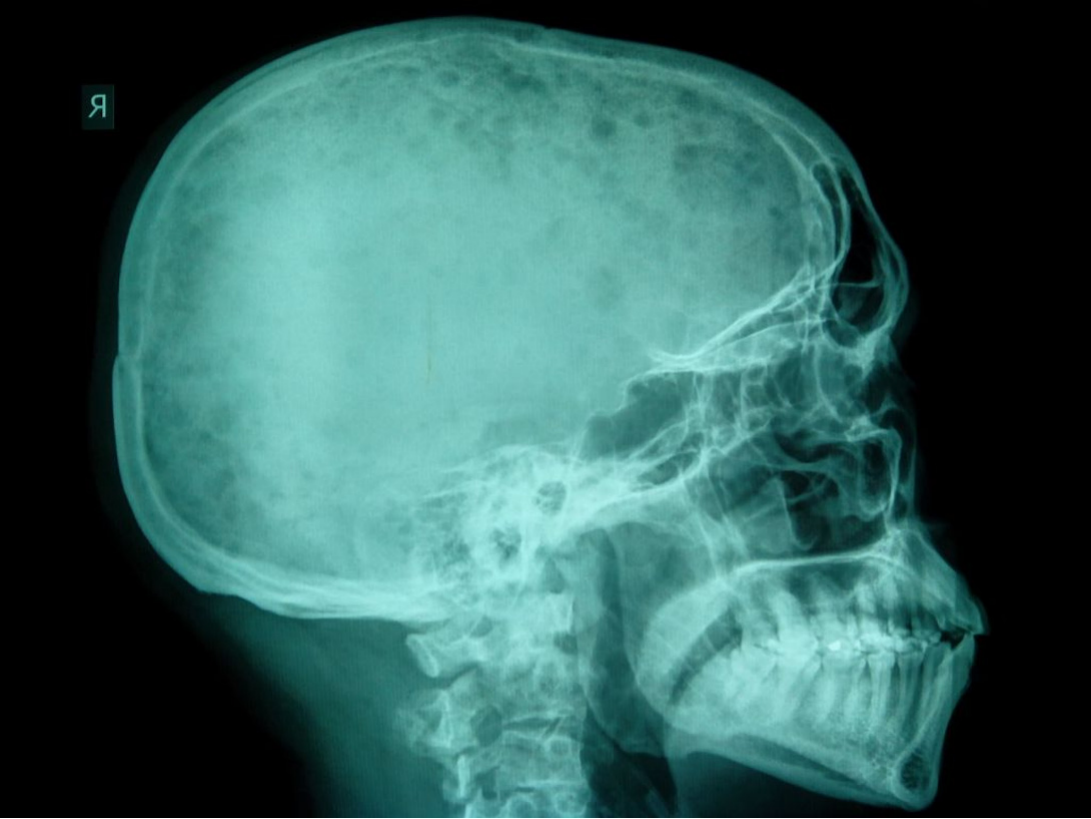
Lateral plain radiograph of the skull showing diffuse osteolytic lesions scattered all over the calvarium

**Figure 2:**
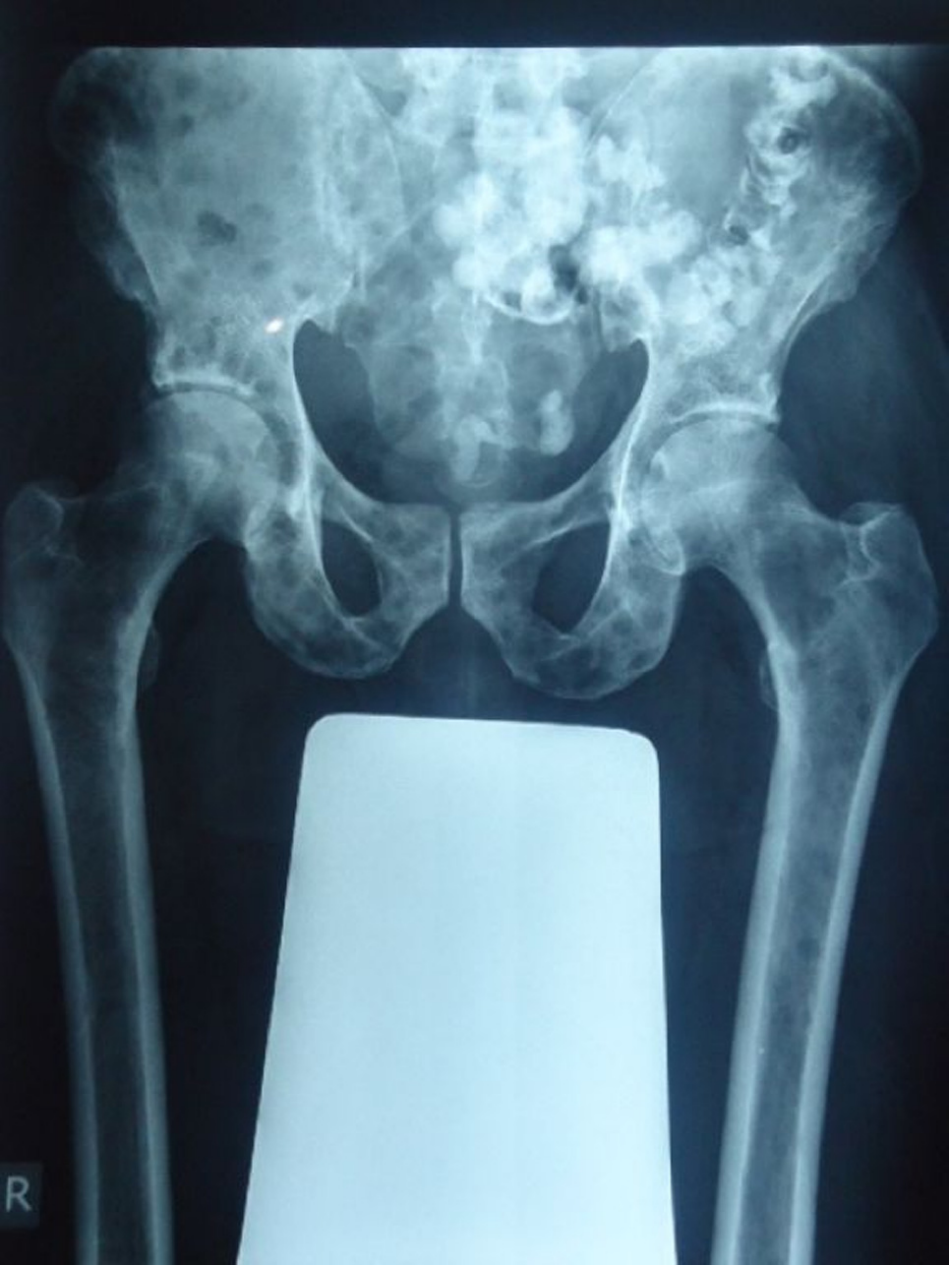
Anteroposterior plain radiograph of the pelvis and proximal portions of the femurs showing diffuse osteolytic lesions over all the demonstrated bones. The radio‐opaque material in the distal bowel loops is residual rectal contrast medium from the previous abdominal CT examination

**Figure 3:**
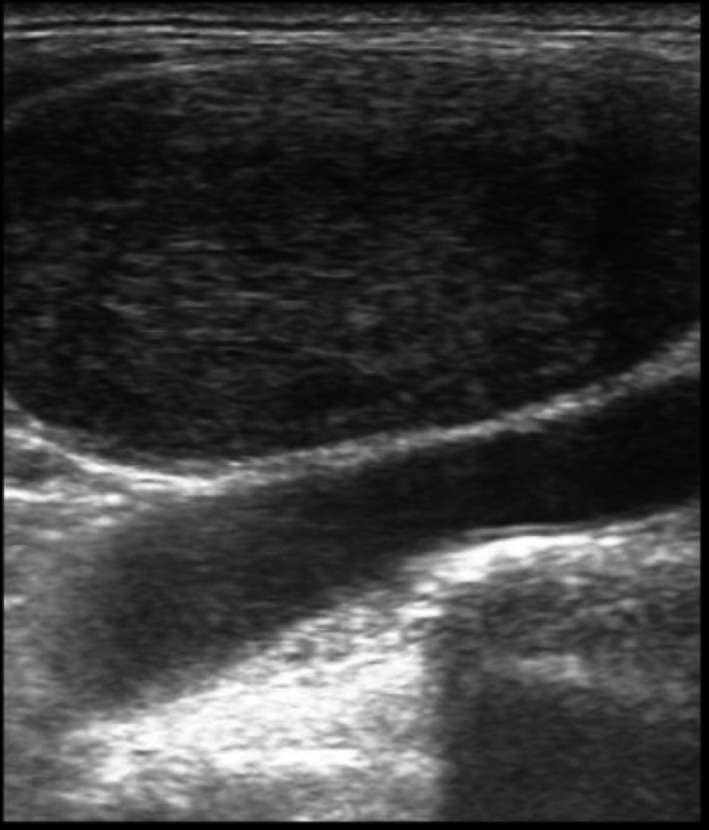
Sonography of the left supraclavicular swelling showing an oval, hypoechoic enlarged lymph node with effaced central hilum. The brachiocephalic vessel and cortex of the left clavicle are seen posteriorly

Plain radiographs (skeletal survey) of the skull, pelvis (Figs. 1 and 2), and lumbosacral spine showed diffuse, osteolytic lesions. No associated periosteal reaction or pathological fracture. Sonography revealed an enlarged prostate gland with irregular outline, and a heterogeneous, nodular parenchymal echotexture. The right kidney was hydronephrotic. Transrectal ultrasound demonstrated a vascularized, heterogeneous nodule in the peripheral zone of the enlarged prostate gland with a bulging capsular outline. Left supraclavicular sonography revealed an oval, hypoechoic mass (enlarged lymph node) with effaced central hilum (Fig. 3).

**Figure 4:**
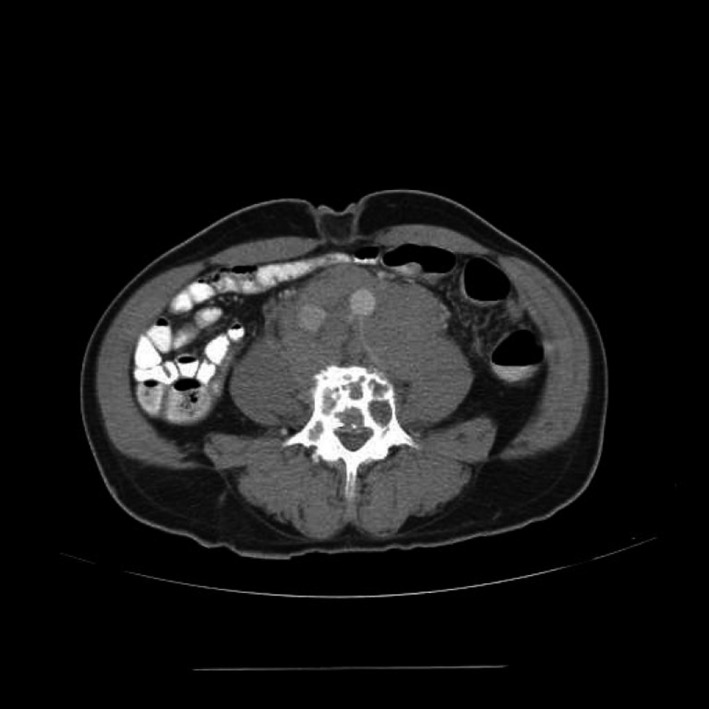
Contrast‐enhanced axial CT of the abdomen showing extensive retroperitoneal adenopathy encasing the abdominal aorta and inferior vena cava, displacing them anteriorly**.** Multiple lytic lesions are seen on the lumbar vertebra body

**Figure 5:**
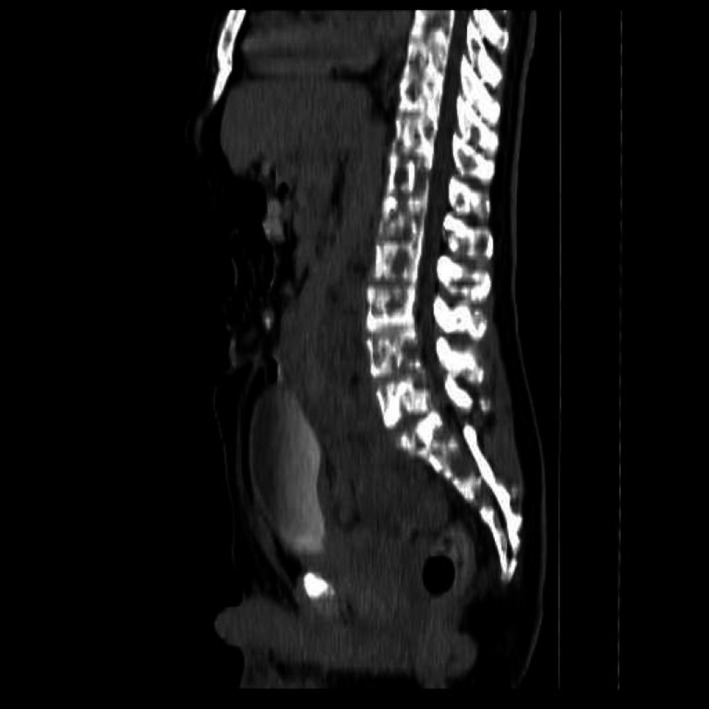
Sagittal reformatted CT of the spine showing extensive lytic lesions/hypodensities on virtually all the demonstrated bony outlines

**Figure 6:**
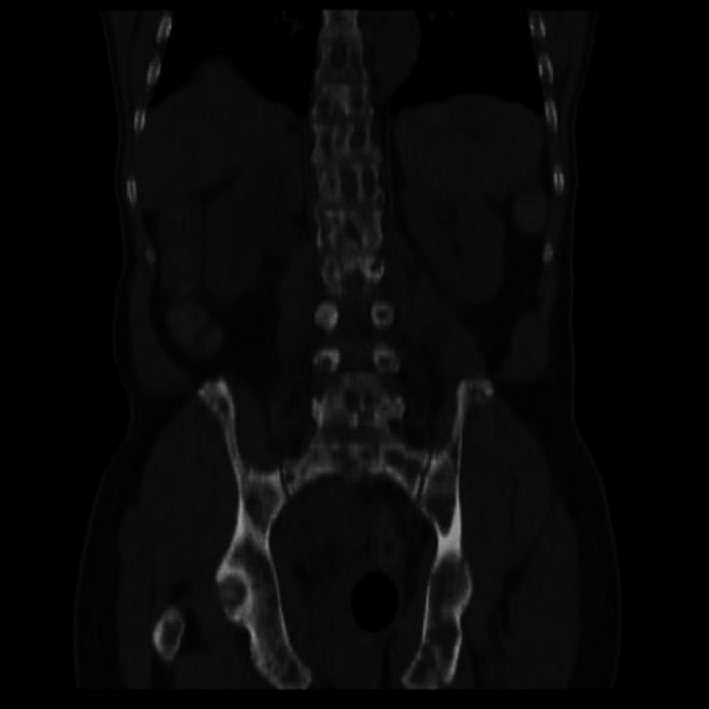
Coronal reformatted CT showing extensive lytic lesions/hypodensities on the demonstrated bones

Abdominopelvic CT with multiplanar reformats showed extensive retroperitoneal adenopathy encasing the abdominal aorta and inferior vena cava, displacing them anteriorly (Fig. 4). The ureters are displaced laterally. The adenopathy extends to the porta hepatis and the right renal hilum, causing right hydronephrosis and intrahepatic biliary ductal dilatation, respectively. The diffuse osteolytic bone lesions (Figs. 5 and 6) and the prostatic enlargement were also visualized.

Bone scan could not done for the patient because the radionuclide imaging facility was unavailable in our institution.

An assessment of prostate carcinoma with nodal and skeletal metastases was made. Serum protein electrophoresis was normal (No M band or spike in the globulin region). Bone marrow aspiration was also within normal limits (Plasma cells constituted 1% of bone marrow cells; normocellular/normoblastic picture).

Prostate cancer cells were seen in the excisional biopsy sample of the supraclavicular adenopathy, and the prostatic tissue biopsy sample showed poorly differentiated infiltrating adenocarcinoma (Gleason score of 5 + 3  = 8).

The patient underwent bilateral total orchidectomy. The testes did not contain malignant cells on histology. He was also placed on antiandrogen tablet (Flutamide). He responded well to treatment and has been on follow‐up at the urologic clinic for 18 months since diagnosis. The clinical symptoms have largely resolved without onset of new ones. The supraclavicular lymphadenopathy has also regressed. However, the osteolytic lesions are still present on repeat plain radiographs but are stable. Unfortunately, repeat staging with cross‐sectional imaging has not been possible due to financial constraint.

Informed consent to report this case was obtained from the patient.

## Discussion

This patient had two uncommon presentations of prostate carcinoma—diffuse osteolytic metastases 1, 2 and supraclavicular lymphadenopathy 3. Supraclavicular lymphadenopathy is commoner with kidney and breast cancers 3. Bone metastases occur in up to 90% of patients with late/advanced stage of prostate cancer while approximately 30% of patients have bone metastasis at the time of diagnosis 4. Osteolytic secondaries from the prostate cancer are usually solitary 5.

This patient presented with bone pains which necessitated a skeletal survey. The main differential diagnoses of the radiographic appearance were diffuse osteolytic bone metastasis from prostate cancer and multiple myeloma. Apart from sending osteolytic deposits to bones, the two disease entities share other similarities such as age of presentation, being more common in Blacks and in men (Though prostate cancer is a male disease, other cancers can also cause diffuse bone osteolysis), elevated ESR, and bone pain/back pain. However, the main challenge in evaluation is how to conclusively distinguish the two entities as their treatment approaches differ. This can be achieved by adopting a methodical approach to investigating the patient using biochemical and histological tests. Anemia, leukopenia, thrombocytopenia, hypercalcemia, and Bence‐Jones proteinuria (Proteinuria greater than 1 g of protein in 24‐hour urine collection) are prominent features of multiple myeloma but not in diffuse osteolytic metastases. Furthermore, derangement in the level and proportion of serum proteins (Albumin and globulin), M band or a spike in the globulin region on serum protein electrophoresis, and bone marrow aspiration showing plasma cells of multiple myeloma should help establish or exclude a diagnosis of multiple myeloma as seen in this case.

Diffuse osteolytic metastasis from prostate cancer is quite rare, with very few cases reported in literature 1, 2, 6, 7, 8, 9, 10. It affected the skull, vertebrae, pelvic bones, ribs, and femurs of this patient which is largely consistent with previously established preferred bony secondary sites of spine, ribs, and femur in that order 11. Compared to previous cases 1, 2, 6, 7, 9, 10, the spread of lesions this patient is relatively more extensive. Furthermore, the PSA value is much lower (16 ng/ml) than that of the similar cases reported earlier (range = 100–7242 ng/mL) 1, 5, 8, 10. This finding appears counterintuitive considering the extent of disease. Such PSA level ‐disease burden discordance should reasonably arouse a suspicion of the presence of atypical cytological variants like neuroendocrine differentiation on prostate biopsy. However, the prostate biopsy result in the index patient showed poorly differentiated infiltrating adenocarcinoma and no other atypical variants. It is pertinent to note that other researchers have made a similar observation previously that serum PSA level may even be normal in bone and bone marrow metastases of prostatic adenocarcinoma 12.

It is noteworthy that the focal lytic lesions that give the classic radiographic appearance of multiple discrete, lytic, punched‐out, round lesions in multiple myeloma and diffusely metastatic prostate cancer must be at least 5 mm in size to be considered as true abnormalities 13. In addition, at least 30% of cancellous bone must have been lost before skeletal metastases become evident on plain radiographs, which means they are somewhat insensitive to small lytic lesions 14. Detection of skeletal metastases is essential for staging, choice of therapy, and prognostication 15.

Radionuclide imaging could not be performed for the patient because there are no facilities for bone scintigraphy or hybrid Positron Emission Tomography + Magnetic Resonance Imaging or Computerized Tomography (PET/MRI and PET/CT) in our institution. Serum PSA level is said to be a useful guide for determining whether or not bone scintigraphy is indicated 16. In a patient with prostate cancer without bone symptoms and a serum PSA ≤ 20.0 ng/mL, bone scintigraphy is not indicated because it is unlikely to yield any positive information. In patients with a serum PSA > 20.0 ng/mL and in those with symptoms suggestive of bone metastasis (regardless of serum PSA levels), bone scintigraphy is indicated 16.

Lastly, diffuse osteolytic metastases simulating multiple myeloma is not exclusive to prostate cancer. It has been reported with hepatocellular carcinoma 17 and breast cancer 18. Indeed, apart from simulation/mimicry, it must also be borne in mind that multiple myeloma can also occur synchronously or metachronously with these malignancies 19, 20. Therefore, a high index of suspicion must always be maintained, especially where there are other soft tissue masses besides the lytic bone lesions.

In conclusion, diffuse osteolytic metastasis is very rare form of prostate cancer metastases which must be a topmost consideration in elderly male patients with diffuse osteolytic lesions on radiography. It can present like this even when the serum PSA is not markedly elevated. Multiple myeloma needs to be excluded in such cases.

## Author Contributions

BMI: was responsible for conception, literature search/review, manuscript writing, and critical review.

## Conflict of Interest

None declared.
